# Exploring the tolerable region for HiBiT tag insertion in the hepatitis B virus genome

**DOI:** 10.1128/msphere.00518-24

**Published:** 2024-09-30

**Authors:** Asako Murayama, Hitomi Igarashi, Norie Yamada, Hussein Hassan Aly, Masaaki Toyama, Masanori Isogawa, Tetsuro Shimakami, Takanobu Kato

**Affiliations:** 1 Department of Virology II, National Institute of Infectious Diseases, Tokyo, Japan; 2 Department of Gastroenterology, Graduate School of Medicine, Kanazawa University, Ishikawa, Japan; University of Michigan, Ann Arbor, Michigan, USA

**Keywords:** HBV, preS1, luciferase, NTCP, primary hepatocyte

## Abstract

A cell culture system that allows the reproduction of the hepatitis B virus (HBV) life cycle is indispensable to exploring novel anti-HBV agents. To establish the screening system for anti-HBV agents, we exploited the high affinity and bright luminescence (HiBiT) tag and comprehensively explored the regions in the HBV genome where the HiBiT tag could be inserted. The plasmids for the HiBiT-tagged HBV molecular clones with a 1.38-fold HBV genome length were prepared. The HiBiT tag was inserted into five regions: preS1, preS2, hepatitis B e (HBe), hepatitis B X (HBx), and hepatitis B polymerase (HB pol). HiBiT-tagged HBVs were obtained by transfecting the prepared plasmids into sodium taurocholate cotransporting polypeptide-transduced HepG2 (HepG2/NTCP) cells, and their infectivity was evaluated in human primary hepatocytes and HepG2/NTCP cells. Among the evaluated viruses, the infection of HiBiT-tagged HBVs in the preS1 or the HB pol regions exhibited a time-dependent increase of the hepatitis B surface antigen (HBsAg) level after infection to HepG2/NTCP cells as well as human primary hepatocytes. Immunostaining of the hepatitis B core (HBc) antigen in infected cells confirmed these viruses are infectious to those cells. However, the time-dependent increase of the HiBiT signal was only detected after infection with the HiBiT-tagged HBV in the preS1 region. The inhibition of this HiBiT-tagged HBV infection in human primary hepatocytes and HepG2/NTCP cells by the preS1 peptide could be detected by measuring the HiBiT signal. The infection system with the HiBiT-tagged HBV in HepG2/NTCP cells facilitates easy, sensitive, and high-throughput screening of anti-HBV agents and will be a useful tool for assessing the viral life cycle and exploring antiviral agents.

**IMPORTANCE:**

Hepatitis B virus (HBV) is the principal causative agent of chronic hepatitis. Despite the availability of vaccines in many countries, HBV infection has spread worldwide and caused chronic infection. In chronic hepatitis B patients, liver inflammation leads to cirrhosis, and the accumulation of viral genome integration into host chromosomes leads to the development of hepatocellular carcinoma. The currently available treatment strategy cannot expect the eradication of HBV. To explore novel anti-HBV agents, a cell culture system that can detect HBV infection easily is indispensable. In this study, we examined the regions in the HBV genome where the high affinity and bright luminescence (HiBiT) tag could be inserted and established an HBV infection system to monitor infection by measuring the HiBiT signal by infecting the HiBiT-tagged HBV in sodium taurocholate cotransporting polypeptide-transduced HepG2 (HepG2/NTCP) cells. This system can contribute to screening for novel anti-HBV agents.

## INTRODUCTION

Hepatitis B virus (HBV) infection is a significant cause of chronic liver diseases, including cirrhosis and hepatocellular carcinoma (1). Although effective vaccines for HBV are available, HBV affects approximately 290 million people worldwide (2). Currently, the eradication of HBV cannot be expected, because the covalently closed circular DNA (cccDNA) of HBV in infected hepatocytes is difficult to eliminate by the available treatment strategy (3, 4). Thus, novel anti-HBV agents are urgently needed. To explore novel anti-HBV agents, a cell culture system that allows the reproduction of the HBV life cycle is indispensable. Sodium taurocholate cotransporting polypeptide (NTCP) was identified as an HBV receptor, and NTCP-transduced cell lines derived from hepatocytes contributed to the establishment of such systems (5). In the HBV cell culture systems, HBV infection and replication are monitored by measuring hepatitis B surface antigen (HBsAg), hepatitis B e antigen (HBeAg), and HBV DNA. However, the measurement of these markers requires specialized measurement kits or reagents and is known to be affected by HBV genotypes or mutations in target regions (6). HBV has a partially double-stranded 3.2-kb DNA genome that comprises four open reading frames (ORFs), which encode HBsAg, HBeAg/hepatitis B core antigen (HBcAg), hepatitis B polymerase (HB pol), and hepatitis B X protein (HBx). HBsAg consists of three species: small (S-), middle (M-), and large (L-) HBsAgs. S-HBsAg has 266 amino acids (aa), and M-HBsAg and L-HBsAg have additional regions of preS2 (55 aa) and preS1+preS2 (174 aa), respectively. Recently, we reported a novel monitoring system for HBV infection and replication using the high affinity and bright luminescence (HiBiT) tag (7). In that system, the HiBiT tag was inserted in the N-terminus of the preS1 region of the L-HBsAg. The infection of HiBiT-tagged HBV could be monitored by measuring the HiBiT signal in the culture medium after infection with human primary hepatocytes. However, the generated HiBiT-tagged HBV had lower infection and replication efficiencies than the cell culture-generated HBV (HBVcc) without the HiBiT tag, and infection could not be detected in cultured cells other than human primary hepatocytes. Thus, in this study, we comprehensively explored the regions in the HBV genome where the HiBiT tag could be inserted and evaluated the infectivity of the HiBiT-tagged HBV in the established NTCP-transduced HepG2 cell line that is reported to be highly susceptible to HBV (8).

## RESULTS

### Generation of HiBiT-tagged HBVcc by plasmid transfection

**Fig 1:**
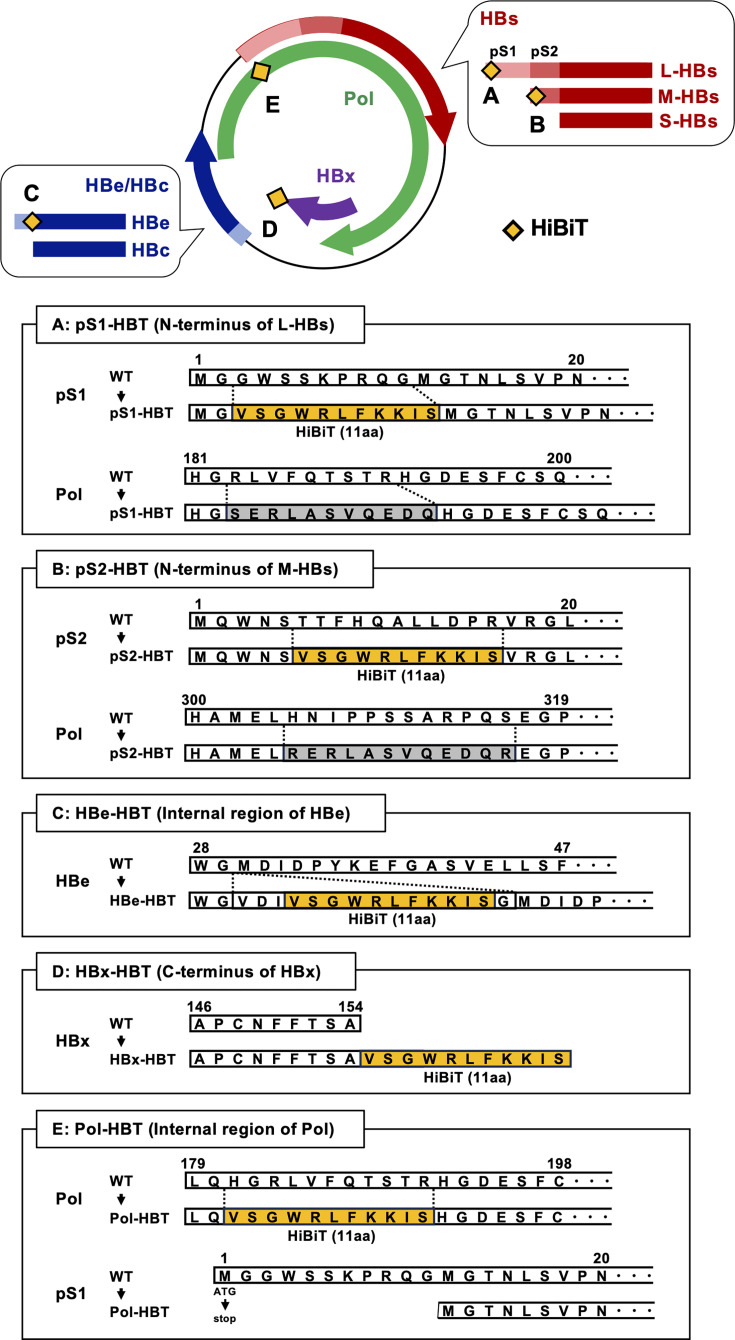
Schematic representation of the HiBiT-tag-inserted region in the HBV molecular clone used in this study. The HiBiT tag was inserted into five regions: the preS1 region (**A**), preS2 region (**B**), HBe region (**C**), HBx region (**D**), and HB pol region (**E**) of the HBV genome. Amino acids of the inserted HiBiT tag (filled with yellow) and the affected HBV region are indicated.

**Fig 2:**
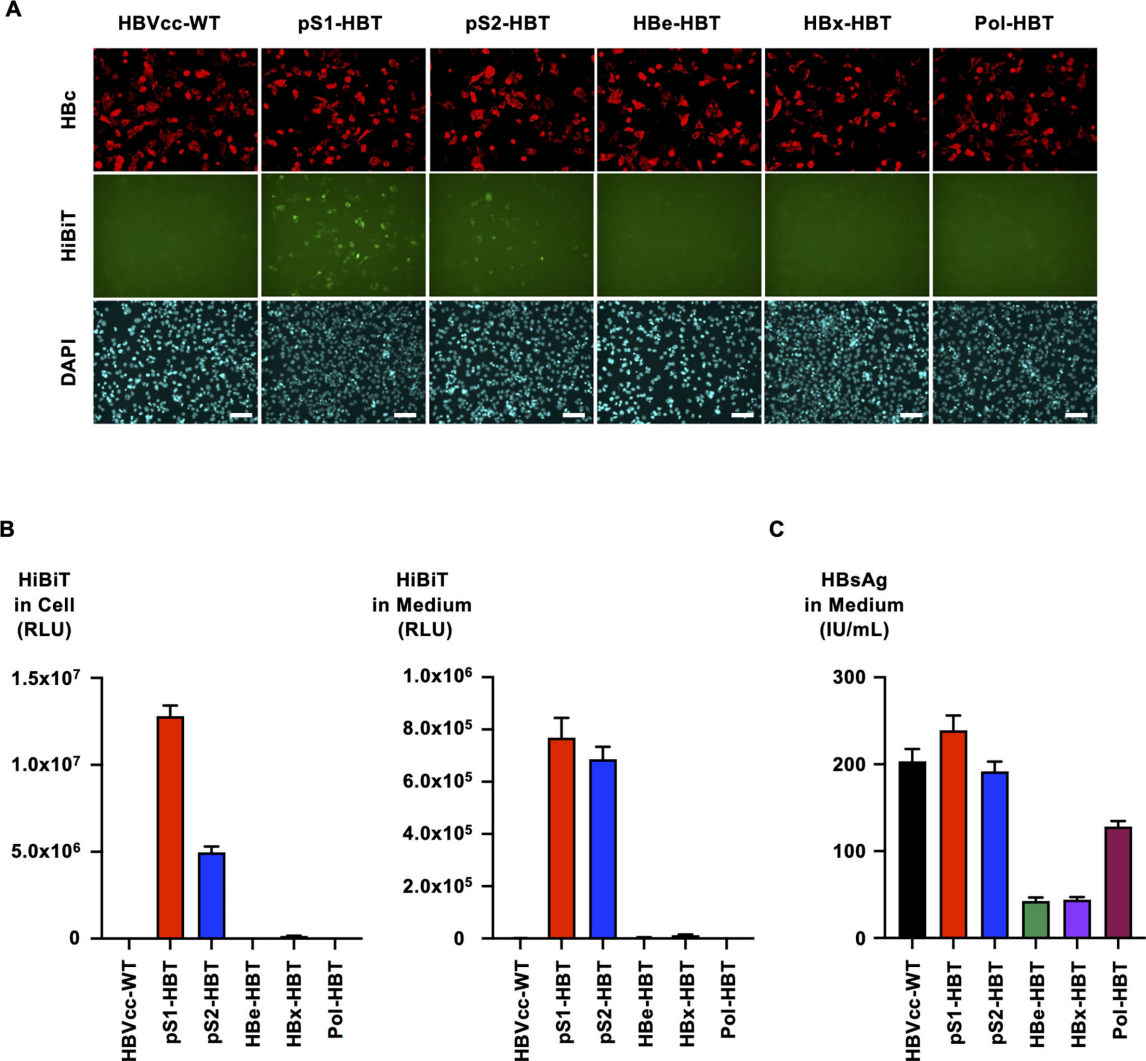
Detection of HBcAg and HiBiT-tagged viral proteins by transfection of HiBiT-tagged HBVcc plasmids. (**A**) The production of HBcAg and HiBiT-tagged viral proteins was detected by immunostaining with anti-HBc and anti-HiBiT antibodies 3 days after transfection. Nuclei were visualized by staining with DAPI. The white bar indicates 100 µM. (**B**) The HiBiT signals in cells and culture medium were measured after transfection of plasmids for HiBiT-tagged HBVcc. HBVcc-WT was used as a negative control. (**C**) HBsAg production in the culture medium was measured after transfection of plasmids for HiBiT-tagged HBVcc.

To produce HiBiT-tagged HBVcc, the HiBiT tag sequence was inserted into five regions (preS1, preS2, HBe, HBx, and HB pol) of a replication-competent molecular clone of HBV (genotype C) generating plasmids of pS1-HBT, pS2-HBT, HBe-HBT, HBx-HBT, and Pol-HBT (Fig. 1). To assess the influence of the HiBiT tag insertion to these regions, the expression of HiBiT-tagged HBV proteins was detected by immunostaining after transfection of five kinds of plasmids for HiBiT-tagged HBVcc into NTCP-transduced HepG2 (HepG2/NTCP) cells. The plasmid of HBVcc without the HiBiT tag (HBVcc-WT) was also used as a control. Although the number of HBcAg-positive cells suggested the transfection efficiencies were similar among these plasmid-transfected cells, the expression of HiBiT-tagged viral protein was detected only in pS1-HBT- and pS2-HBT-plasmid-transfected cells (Fig. 2A). The staining intensity of the HiBiT tag was greater in the pS1-HBT-plasmid-transfected cells than in the pS2-HBT-plasmid-transfected cells. The HiBiT signals in these plasmid-transfected cells were also evaluated. As reflected by the staining results of the HiBiT-tagged HBV proteins, the HiBiT signals in the pS1-HBT- and pS2-HBT-plasmid-transfected cells were detectable, although the HiBiT signals were very low in the cells transfected with plasmids for other clones (Fig. 2B). The HiBiT signals were also detected in the culture media of pS1-HBT- and pS2-HBT-plasmid-transfected cells. To assess the influence of the HiBiT tag insertion on the production of HBV protein, HBsAg levels in the culture media of these plasmid-transfected cells were measured (Fig. 2C). The HBsAg levels of pS1-HBT- and pS2-HBT-plasmid-transfected cells were similar to those of HBVcc-WT. The HBsAg level in the culture medium of Pol-HBT-plasmid-transfected cells was also high and close to that in the culture medium of HBVcc-WT, but the levels of HBe-HBT- and HBx-HBT-plasmid-transfected cells were substantially lower than those in the culture media of the other groups. HBeAg was detected only in the culture medium of HBe-HBT-plasmid-transfected cells (data not shown). To clarify whether the functions of HB polymerase and HBc in HiBiT-tagged HBVcc were maintained, plasmids of HiBiT-tagged HBVcc were transfected into HepG2/NTCP cells, which were then treated with entecavir (ETV). The HBV DNA in the culture medium was measured by real-time PCR. Although the level of HBV DNA in the culture medium differed among the HiBiT-tagged HBVcc, ETV treatment suppressed the release of encapsidated HBV DNA of HiBiT-tagged HBVcc into the culture medium as well as HBVcc-WT, suggesting that the functions of HB polymerase and HBc were maintained (Fig. S1).

### Purification of HiBiT-tagged HBVcc by iodixanol density gradient

**Fig 3:**
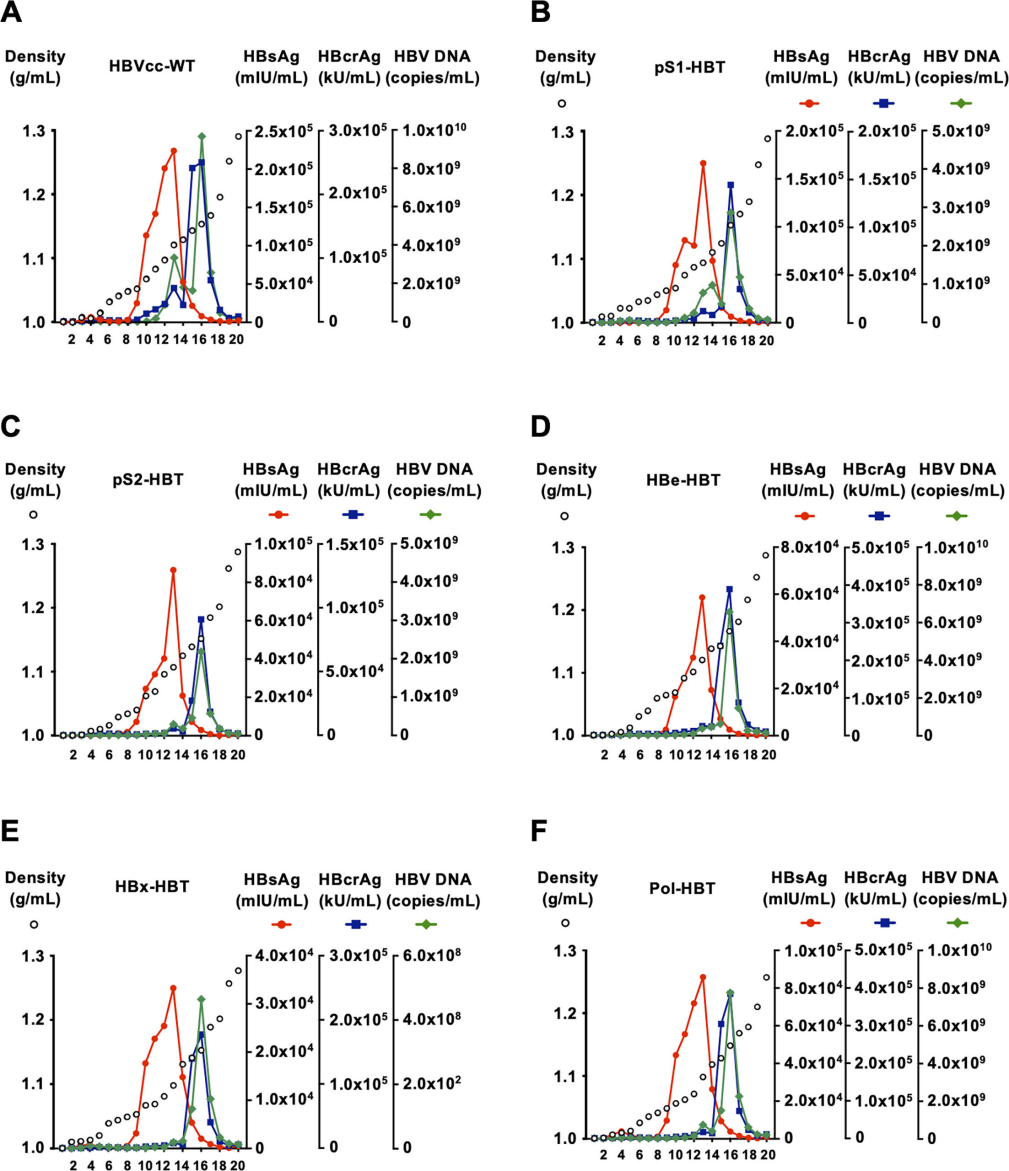
Iodixanol density gradient analysis of HBVcc-WT and HiBiT-tagged HBVcc. HBVcc-WT (**A**) and HiBiT-tagged HBVcc of pS1-HBT (**B**), pS2-HBT (**C**), HBe-HBT (**D**), HBx-HBT (**E**), and Pol-HBT (**F**) in the culture media of transfected cells were concentrated and purified by iodixanol density gradients. The titers of HBsAg and HBcrAg were measured in each fraction, and the HBV DNA concentration was quantified by real-time PCR after treatment with DNase.

To prepare the inoculum of HiBiT-tagged HBVcc, the culture medium was collected from HepG2/NTCP cells that were transfected with the plasmids of HiBiT-tagged HBVcc. The harvested HiBiT-tagged HBVcc was analyzed in an iodixanol density gradient, and the influence of the HiBiT tag insertion was evaluated. In the density gradient profile of HBVcc-WT, HBsAg has a single peak at fractions 13 (Fig. 3). Hepatitis B core-related antigen (HBcrAg) has a major peak at fraction 16 and a minor peak at fraction 13. There were two peaks of HBV DNA at fractions 13 and 16. The peak of infectivity was detected in fraction 14 (Fig. S2). Similar profiles were observed in the density gradients of other clones of HiBiT-tagged HBVcc, albeit with varying levels of HBsAg, HBcrAg, and HBV DNA (Fig. 3). Therefore, the peak fractions of infectivity in the density gradients of other HiBiT-tagged HBVcc were expected in fraction 14, and these fractions were used for further experiments.

### Infection of HiBiT-tagged HBVcc in human primary hepatocytes

**Fig 4:**
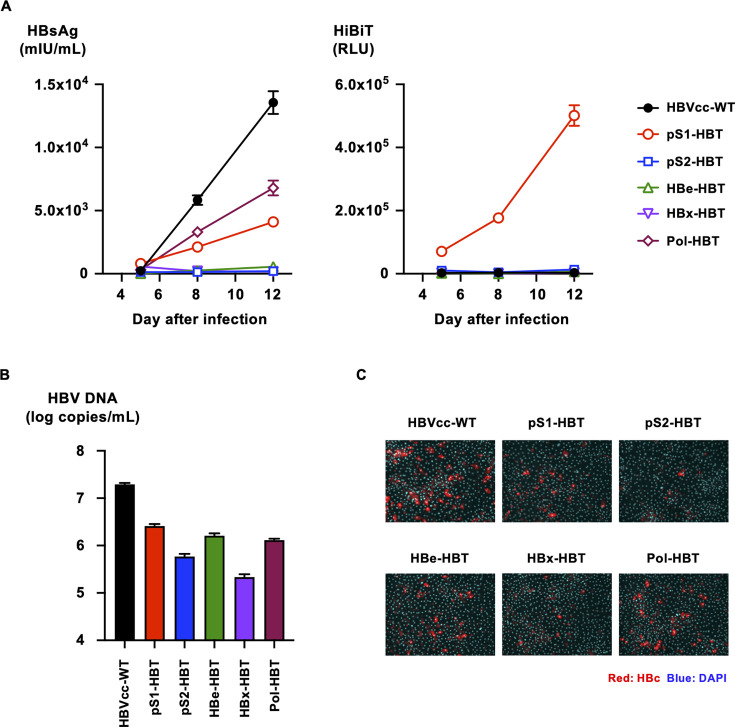
Infection of HiBiT-tagged HBVcc in human primary hepatocytes. (**A**) Human primary hepatocytes were infected with HiBiT-tagged HBVcc at 200 GEq/cell, and the infection efficiencies were compared with those of HBVcc-WT by monitoring HBsAg and HiBiT signals in culture medium on the indicated days after infection. (**B**) The HBV DNA levels in the culture media of HBVcc-WT- and HiBiT-tagged HBVcc-infected cells were assessed by real-time PCR with a primer and probe set designed to target the HBs region after treatment with DNase after 12 days of culture. (**C**) HBVcc-WT- and HiBiT-tagged HBVcc-infected cells were detected by staining with rabbit polyclonal anti-HBc antibody and Alexa Fluor 555-conjugated anti-rabbit IgG. Nuclei were visualized by staining with DAPI.

To assess the infectivity of HiBiT-tagged HBVcc, the generated and purified viruses were used to infect human primary hepatocytes at 200 genome equivalents (GEq) per cell, and the infection efficiencies were compared by monitoring HBsAg and HiBiT signals in culture medium. A time-dependent increase in HBsAg was observed after infection with HBVcc-WT (Fig. 4A, left panel). Similar but low-level increases in HBsAg were also observed after infection with Pol-HBT and pS1-HBT. The increase in HBsAg was not apparent in other HiBiT-tagged HBVcc infections. A time-dependent increase in the HiBiT signal was observed after pS1-HBT infection, although it was scarcely detected in other HiBiT-tagged HBVcc infection (Fig. 4A, right panel). The HBV DNA levels in the culture media of these HiBiT-tagged HBVcc-infected cells were also assessed after 12 days of culture. The highest level of HBV DNA in the culture medium was detected after infection with HBVcc-WT, followed by infection with pS1-HBT, HBe-HBT, and Pol-HBT (Fig. 4B). The HBV DNA levels after infection with pS2-HBT and HBx-HBT were the lowest and were 2-log-fold lower than those with HBVcc-WT. At 12 days after infection, HBcAg in infected cells was also detected to confirm infection. Similar to the HBV DNA data, the highest number of infected cells was observed after infection with HBVcc-WT, followed by infection with pS1-HBT, HBe-HBT, and Pol-HBT (Fig. 4C). A limited number of infected cells were detected by infection with pS2-HBT and HBx-HBT. The expression of HiBiT-tagged viral proteins was not detected in HBcAg-positive cells by immunostaining with an anti-HiBiT antibody (data not shown), although the maintenance of the inserted HiBiT tag in the infected HBV was confirmed by sequencing of the HiBiT-tagged HBV in the culture medium at 12 days after infection (data not shown).

### Infection of HiBiT-tagged HBVcc in HepG2/NTCP cells

**Fig 5:**
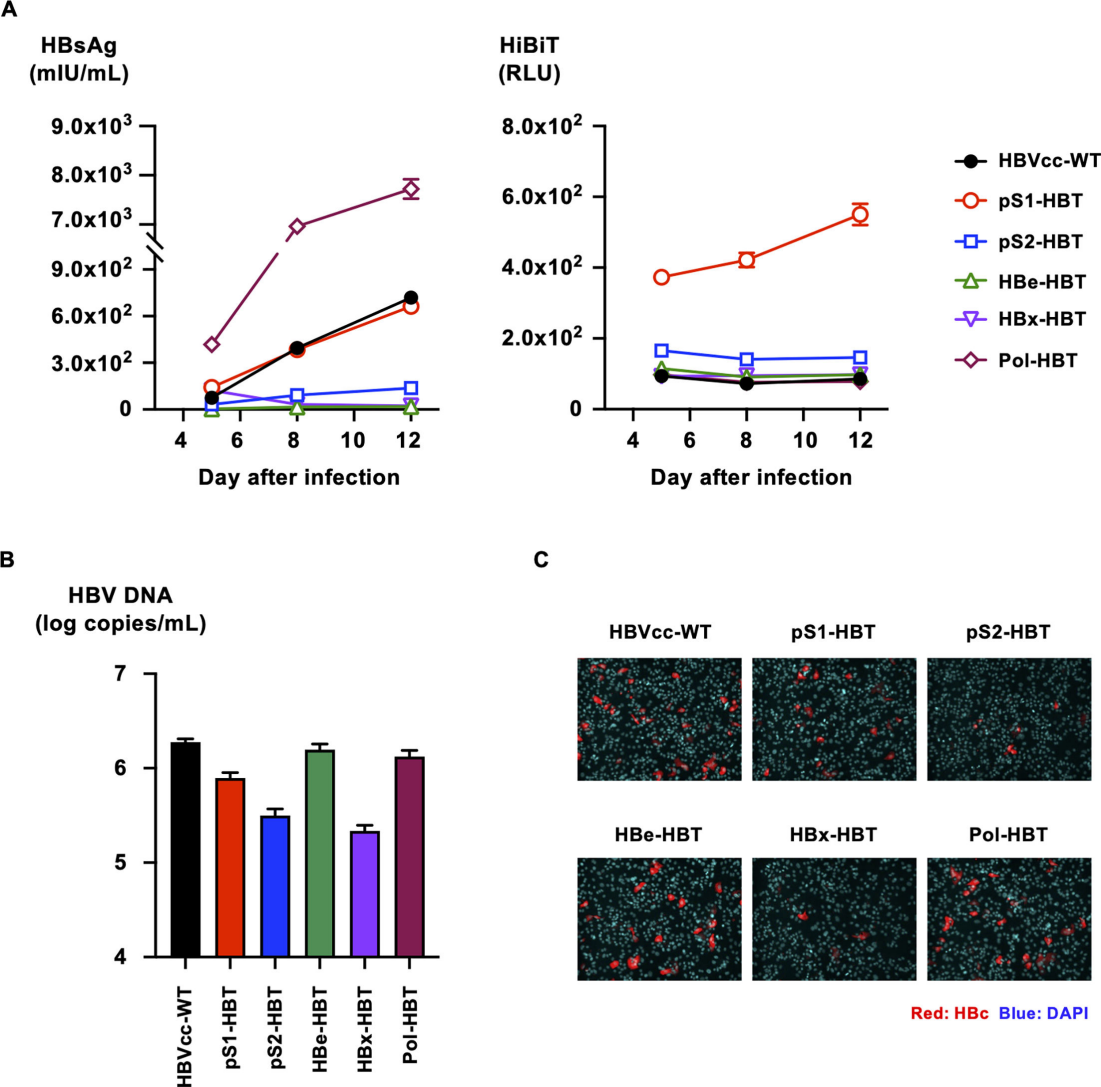
Infection of HepG2/NTCP cells with HiBiT-tagged HBVcc.(**A**) HepG2/NTCP cells were infected with HiBiT-tagged HBVcc at 200 GEq/cell, and the infection efficiencies were compared with those of HBVcc-WT by monitoring HBsAg and HiBiT signals in the culture media on the indicated days after infection. (**B**) The HBV DNA levels in the culture media of HBVcc-WT- and HiBiT-tagged HBVcc-infected cells were assessed by real-time PCR with a primer and probe set designed to target the HBs region after treatment with DNase after 12 days of culture. (**C**) HBVcc-WT- and HiBiT-tagged HBVcc-infected cells were detected by staining with rabbit polyclonal anti-HBc antibody and Alexa Fluor 555-conjugated anti-rabbit IgG. Nuclei were visualized by staining with DAPI.

HiBiT-tagged HBVcc infection was also assessed in HepG2/NTCP cells. HiBiT-tagged HBVcc was infected at 200 GEq/cell, and the levels of HBsAg and HiBiT signals were monitored. Similar to infection in human primary hepatocytes, a time-dependent increase in HBsAg was observed after infection with HBVcc-WT, although the HBsAg level was approximately 20-fold lower than that after infection in human primary hepatocytes (Fig. 5A, left panel). In contrast to infection in human primary hepatocytes, a greater increase in HBsAg was observed after infection with Pol-HBT in comparison with that after infection with HBVcc-WT. A similar increase in HBsAg to the infection with HBVcc-WT was observed after infection of pS1-HBT. An increase in HBsAg was not apparent after infection with other HiBiT-tagged HBVcc. A time-dependent increase in the HiBiT signal was observed only after infection with pS1-HBT (Fig. 5A, right panel). The HBV DNA levels in the culture media after infection with HBVcc-WT, HBe-HBT, and Pol-HBT were comparable. The level of HBV DNA after infection with pS1-HBT was slightly lower than that with HBVcc-WT. The levels of HBV DNA after infection with pS2-HBT and HBx-HBT were 10-fold lower than that with HBVcc-WT (Fig. 5B). Similar to the HBV DNA data, the highest numbers of infected cells were observed after infections with HBVcc-WT, HBe-HBT, and Pol-HBT (Fig. 4C). The infection was also detectable after infection with pS1-HBT, but the number of infected cells was low. Limited numbers of infected cells were detected by infection with pS2-HBT and HBx-HBT. Similar to the infection in human primary hepatocytes, HiBiT-tagged viral proteins were not detected by immunostaining with an anti-HiBiT antibody in any HiBiT-tagged HBVcc-infected cells (data not shown), although the maintenance of the inserted HiBiT tag in the infected HBV was confirmed by sequencing the infected HiBiT-tagged HBVcc (data not shown). To confirm the infection of HiBiT-tagged HBVcc, cccDNA productions in infected cells were evaluated. As well as HBVcc-WT infected cells, cccDNA was detected in HiBiT-tagged HBVcc-infected cells, although the detected cccDNA titers were 1- to 1.5-log-fold lower than that in HBVcc-WT infected (Fig. S3).

### Inhibition of HiBiT-tagged HBVcc infection by the preS1 peptide

**Fig 6:**
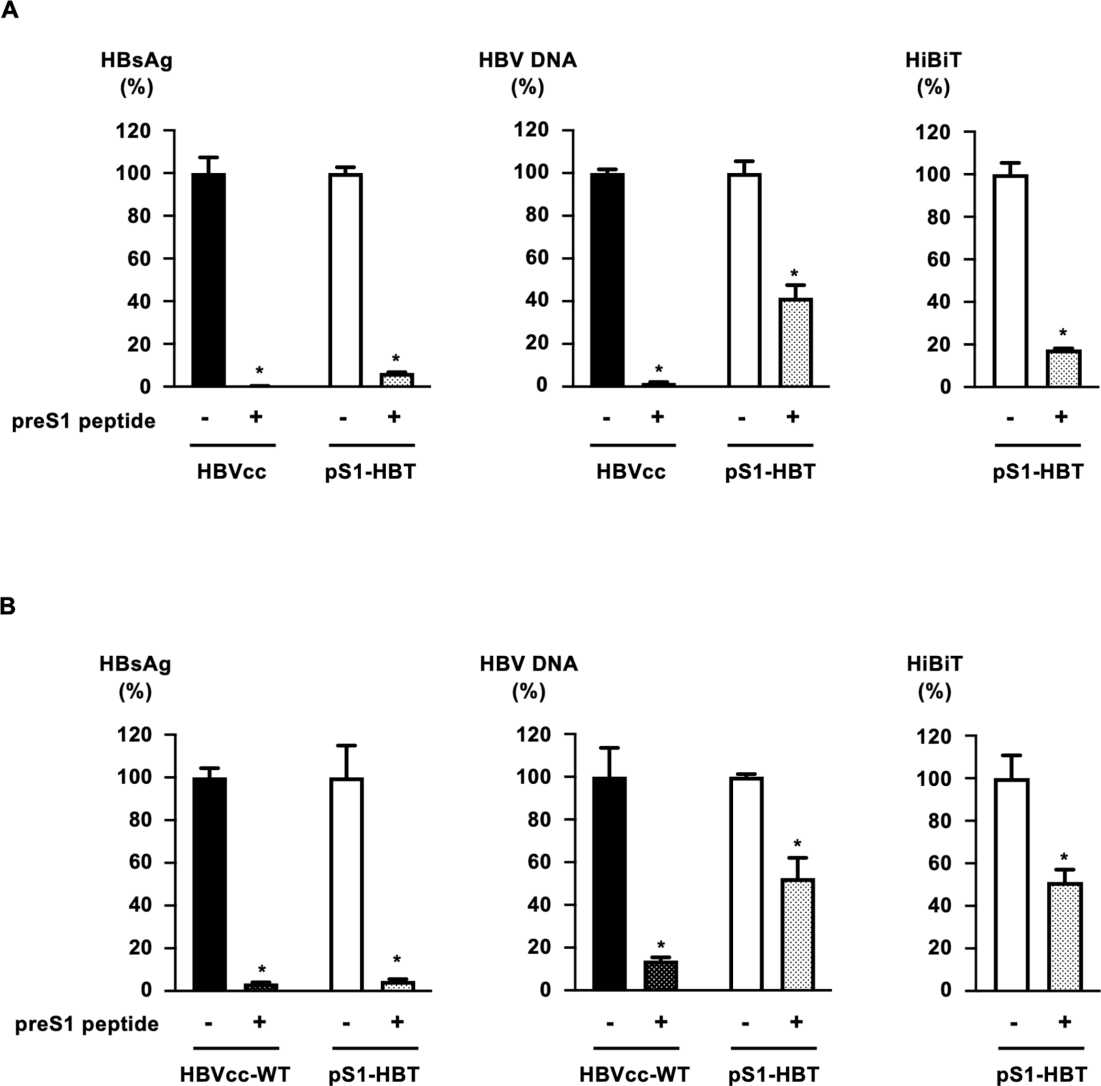
Inhibition of HiBiT-tagged HBVcc infection by the preS1 peptide. HBVcc-WT and pS1-HBT were mixed with the preS1 peptide at a concentration of 200 nM, and human primary hepatocytes (**A**) and HepG2/NTCP cells (**B**) were infected at 200 GEq/cell. The levels of HBsAg and HBV DNA in culture media were measured 12 days after infection. The HiBiT signal in the culture medium was also quantified in the pS1-HBT-infected cells. *P < 0.05 compared with the infection without preS1 peptide group.

To clarify the usefulness of HiBiT-tagged HBVcc for the screening of anti-HBV agents, we examined the inhibitory effect of the preS1 peptide on the infection with HiBiT-tagged HBVcc onto human primary hepatocytes and HepG2/NTCP cells. The preS1 peptide is known to inhibit the interaction of the preS1 region of HBV with NTCP. In this study, pS1-HBT, which showed an increase in the HiBiT signal after infection in primary human hepatocytes and HepG2/NTCP cells, was used and compared with HBVcc-WT. HBVcc-WT and pS1-HBT were mixed with the preS1 peptide at a concentration of 200 nM and were used to infect human primary hepatocytes at 200 GEq/cell. PreS1 peptide treatment significantly reduced HBsAg and HBV DNA levels in the culture medium on day 12 after infection with HBVcc-WT (Fig. 6A). A significant decrease in the HiBiT signal after pS1-HBT infection was also detected after preS1 peptide treatment. Similarly, when infected in HepG2/NTCP cells, reduced levels of HBsAg, HBV DNA, and HiBiT signals by preS1 peptide treatment were observed (Fig. 6B).

## DISCUSSION

An HBV cell culture system enables the reproduction of the HBV life cycle in cell culture and contributes to investigations of viral characteristics and antiviral agents. The insertion of appropriate tags in the viral genome and the monitoring of viral infection and replication by measuring the production of tagged viral proteins make such investigations easier and more inexpensive. In this study, by using a small HiBiT tag of 11 aa, we explored the region suitable for tag insertion in the HBV genome. We selected five regions in four ORFs, generated five kinds of HiBiT-tagged HBVcc, and evaluated the infection and replication efficiencies in both human primary hepatocytes and HepG2/NTCP cells. Among the evaluated viruses, pS1-HBT, in which the HiBiT tag was inserted into the N-terminus of the preS1 region, can be used to monitor infection and replication in HepG2/NTCP cells as well as in human primary hepatocytes by measuring the HiBiT signal.

By transfecting the plasmids for HiBiT-tagged HBVcc, the HiBiT signals in cells and culture media were detected only in pS1-HBT and pS2-HBT. Furthermore, although transfection efficiencies were comparable among the HiBiT-tagged HBVcc clones, HiBiT-tagged viral proteins were detected only in pS1-HBT- and pS2-HBT-transfected cells by immunostaining. These data suggest that the HiBiT tags inserted in other HiBiT-tagged HBVcc were not functional or that the expression levels of the tagged proteins of other HiBiT-tagged HBVcc were not enough for detection. The HiBiT tag insertion regions of pS1-HBT and pS2-HBT were both in the N-termini of the inserted proteins, and the other regions were either internal regions or the C-terminus. Thus, the N-terminal insertion of the HiBiT tag may be more functional in the case of HBV proteins. On the other hand, the production of HBV proteins in which the HiBiT tag was not inserted was also affected by the insertion of the HiBiT tag into other HBV proteins. The levels of HBsAg produced by the transfection of pS1-HBT and pS2-HBT plasmids were comparable to those produced by the transfection of the HBVcc-WT plasmid. In contrast, HBsAg production was slightly reduced in Pol-HBT-plasmid-transfected cells and severely low in HBe-HBT- and HBx-HBT-plasmid-transfected cells. To establish an efficient infection system with tagged HBVcc, selecting the appropriate region that does not influence the production of HBV proteins will be essential.

In the infection study of generated HiBiT-tagged HBVcc, time-dependent increases in HBsAg and HiBiT signals were only observed after infection with pS1-HBT in both human primary hepatocytes and HepG2/NTCP cells. HBV DNA levels in the culture medium of infected cells or the number of infected cells detected by anti-HBc staining was comparable to those of HBVcc-infected cells. Although the maintenance of the inserted HiBiT tag was confirmed by sequencing the infected pS1-HBT, the expression of HiBiT-tagged L-HBsAg was not detected by anti-HiBiT staining in HBcAg-positive cells after infection with pS1-HBT. This finding may suggest that the sensitivity of anti-HiBiT antibodies is lower than that of anti-HBc antibodies.

Pol-HBT exhibited a strong increase of HBsAg beyond the level of HBVcc infection when infected in HepG2/NTCP cells, although this increase was not pronounced when infected in human primary hepatocytes. In this clone, the HiBiT tag was not only inserted into the HB polymerase region but also affected L-HBsAg because of the deletion of methionine at the distal end of the preS1 region. This deletion of methionine resulted in the deletion of 11 amino acids from the N-terminus of L-HBsAg, resulting in the production of only short L-HBsAg, which has been reported to increase HBV infectivity (9). These data suggest that the insertion of a HiBiT tag into the HB polymerase region does not ruin the production of infectious HBV particles, but rather enhances the infectivity of the HBV produced; unfortunately, the inserted HiBiT tag is not detectable. The insertion of the HiBiT tag at the internal region of HB pol may be the reason why this tag is not functional. Another tag that works as a loop structure and can be detected when inserted in the internal region of the target protein may facilitate the establishment of a tag-inserted HBV with high infectivity and replication efficiency that can be monitored by measuring the tagged protein.

When HBe-HBT was infected in human primary hepatocytes and HepG2/NTCP cells, HBsAg production was low, and the HiBiT signal was undetectable. The level of HBsAg produced by plasmid transfection of this clone was also lower compared with that of HBVcc. These data may suggest that the insertion of the HiBiT tag at the HBe region or an increase in genome size reduces HBsAg production. However, the infection of HBe-HBT exhibited a comparable level of HBV DNA in the culture medium and a comparable number of infected cells to the number of HBVcc-infected cells after 12 days of culture. These data suggest that the insertion of the HiBiT tag into this region does not impede the production of infectious HBV particles. An HA-tag insertion into this region is known to produce infectious viral particles, and the infection and replication of HBV can be monitored by measuring the HA-tagged protein (10). Therefore, for this region, only the HiBiT tag may not be suitable for monitoring the infection and replication of HBV by measuring the tag signal.

HBx-HBT infection was not detected in either human primary hepatocytes or HepG2/NTCP cells. Low-level production of HBsAg was observed after the HBx-HBT plasmid was transfected into HepG2/NTCP cells, and the production levels of HBsAg and HBV DNA were low when HBx-HBT was used to infect human primary hepatocytes and HepG2/NTCP cells. The HBx protein has been reported to stimulate HBV replication by activating viral transcription, enhancing both viral polymerase activity and pregenomic RNA encapsidation (11). Thus, the HBx protein in HBx-HBT may become nonfunctional by the insertion of the HiBiT tag, and, as a result, the generation of infectious viral particles after plasmid transfection or the viral protein production after infection of this virus might be hampered.

In this study, we observed pS1-HBT infection in HepG2/NTCP cells, which was not possible in our previous study (7). Moreover, in these cells, the inhibition of pS1-HBT infection by the preS1 peptide could also be detected by measuring the HiBiT signal in the culture medium. The HiBiT-tagged HBVcc infection system for the HepG2/NTCP cell line established in this study enables the exploration of anti-HBV agents targeting the HBV infection step more inexpensively and more easily. In this study, the use of the HepG2/NTCP cell line, which is highly susceptible to HBV infection, was the major reason for the successful observation of the pS1-HBT infection in established cell lines other than human primary hepatocytes. The susceptibility to HBV infection will differ among established cell lines even if they are similarly derived from HepG2 cells. Thus, the selection of cell lines for infection with HBVcc will be important for establishing an efficient cell culture system for HBV. By using other highly susceptible cell lines for HBV infection, HiBiT-tagged HBVcc, for which sufficient infection could not be detected by measuring the HiBiT signal in this study, may become available.

In conclusion, the HiBiT tag insertion at the N-terminus of the preS1 region is the most suitable for establishing a HiBiT-tagged HBV that can reproduce the entire viral life cycle by monitoring the HiBiT signal in cell culture. The infection system with the HiBiT-tagged HBV in HepG2/NTCP cells is easy, sensitive, and suitable for high-throughput screening of anti-HBV agents. It is also a useful tool for assessing the viral life cycle and exploring antiviral agents.

## MATERIALS AND METHODS

### Cell culture

HepG2/NTCP cells that designated HepG2-NTCPsec+ cells have been described previously (8). Human primary hepatocytes (PXB cells, PhoenixBio, Hiroshima, Japan) isolated from human primary hepatocyte-transplanted urokinase-type plasminogen activator/severe combined immunodeficient mice (PXB-mice, PhoenixBio) were grown in culture medium provided by the manufacturer (12).

### Plasmid construction

To produce HBVcc, a replication-competent molecular clone of HBV (genotype C, accession number: AB246345) with a 1.38-fold length of the HBV genome was used as described previously (9). The 11-amino-acid HiBiT tag sequence (VSGWRLFKKIS) was inserted into five regions (preS1, preS2, HBe, HBx, and HB pol) of the four ORFs in the HBV genome of this HBV molecular clone, generating pS1-HBT, pS2-HBT, HBe-HBT, HBx-HBT, and Pol-HBT, respectively. In the pS1-HBT plasmid, the HiBiT tag was inserted between two methionine residues at the N-terminus of L-HBsAg. This plasmid has already appeared in previous reports (7). In the pS2-HBT plasmid, the HiBiT tag was inserted five amino acids after the N-terminus of M-HBsAg because these five amino acids have been reported to be indispensable for the production and secretion of infectious HBV particles (13). In the HBe-HBT plasmid, the HiBiT tag was inserted immediately before the HBc region by mimicking the HA-tagged HBe protein reported by Cai et al. (10). To maintain the epsilon structure, nine additional nucleotides were added before the HiBiT tag sequence. In the HBx-HBT plasmid, the HiBiT tag was inserted at the C-terminus of the HBx protein. In the Pol-HBT plasmid, the HiBiT tag was inserted in the reading frame of the HB polymerase, albeit in a region similar to that of pS1-HBT. In this plasmid, the distal methionine of the two methionines of the L-HBs antigen was removed in anticipation of the high infectivity of the generated HBVcc (9). For the plasmids of pS1-HBV and Pol-HBT, as well as pS2-HBT, the HiBiT tag was inserted in the spacer region of the HB polymerase, and the polymerase function was expected to be maintained (14, 15). For other plasmids for HiBiT-tagged HBVcc, the HiBiT-tag-inserted region was out of the reading frame of the HB polymerase. The information on the generated clones, including the HiBiT-tag-inserted region, affected viral proteins, and presumed HBV genome length, is summarized in Table 1.

**TABLE 1:** Regions affected by HiBiT tag insertion

Clone	HiBiT-inserted ORF	Affected protein	Position	Other affected protein	Precore stop mutation^*c*^	HBV genome length (bp)
pS1-HBT	preS1	L-HBs	N-terminus	HB Pol	+	3,221
pS2-HBT	preS2	L-HBs	Internal region	HB Pol	+	3,215
		M-HBs	N-terminus^*a*^			
HBe-HBT	HBe	HBe	Internal region	None	–	3,260
HBx-HBT	HBx	HBx	C-terminus	HBe^*b*^	+	3,248
Pol-HBT	HB Pol	HB Pol	Internal region	L-HBs	+	3,215

^*a*^ The HiBiT tag was inserted after five amino acids of the M-HBs protein.

^*b*^ HBeAg was not expressed from this construct by the introduction of a precore stop mutation.

^*c*^ +, the precore stop mutation (nt. 1896; G to A) is introduced and no HBeAg is produced. –, the codon at the precore stop mutation is wild-type.

### Production of HiBiT-tagged HBVcc

HepG2/NTCP cells were transfected with the five plasmids using Lipofectamine 3000 Reagent (Thermo Fisher Scientific, Waltham, MA, USA) to produce HiBit-tagged HBVcc. The plasmid for HBVcc without the HiBiT tag (HBVcc-WT) was used as a control. The expression of HBcAg in transfected cells was detected by staining with a rabbit polyclonal anti-HBc antibody (Beacle Inc., Kyoto, Japan) and Alexa Fluor 555-conjugated anti-rabbit IgG (Thermo Fisher Scientific). The expression of HiBiT-tagged protein in transfected cells was detected by staining with a mouse monoclonal anti-HiBiT antibody (Promega, Madison, WI, USA) and Alexa Fluor 488-conjugated anti-mouse IgG (Thermo Fisher Scientific). The nuclei were stained with 4′,6-diamidino-2-phenylindole (DAPI). The production of HiBiT-tagged proteins in transfected cells and culture medium was also measured with a Nano Glo HiBiT Lytic Detection System (Promega) and a Nano Glo HiBiT Extracellular Detection System (Promega), respectively. The production of HBsAg and HBeAg in culture medium was also measured by chemiluminescent enzyme immunoassay using commercial assay kits (Lumipulse G1200, Fujirebio, Tokyo, Japan) (6).

To clarify whether the functions of HB polymerase and HBc in HiBiT-tagged HBVcc were maintained, HiBiT-tagged HBVcc plasmids were transfected into HepG2/NTCP cells, and the transfected cells were treated with ETV (LKT Laboratories, Inc., St. Paul, MN, USA) at a concentration of 100 nM for 6 days. The HBV DNA in the culture medium was measured by real-time PCR with a primer and probe set designed to target the HBs region after treatment with DNase (RQ1 RNase-Free DNase, Promega) and DNA extraction with QIAamp DNA Blood Mini Kit (Qiagen, Hilden, Germany).

The generated HiBiT-tagged HBVcc in the culture medium was harvested 1 week later. The collected medium was passed through a 0.45-µm filter to remove cell debris and was concentrated with Amicon Ultra15 centrifugal filter units (100 kDa, Merck Millipore, Tullagreen, Ireland). HBVcc in the concentrated medium was purified by iodixanol density gradient (10%–40%) centrifugation at 38,000 rpm for 16 hours at 4°C in an SW 41 Ti rotor. HBsAg and HBcrAg in each fraction were measured by a Lumipulse G1200 (Fujirebio). The HBV DNA titer in each fraction was also measured by real-time PCR with a primer and probe set designed to target the HBs region after treatment with DNase (RQ1 RNase-Free DNase) and DNA extraction (16). The infectivity of each fraction was evaluated by infecting HepG2/NTCP cells with the same volume of each fraction. The HBsAg production of infected cells was measured 12 days after infection to identify the peak fraction of infectivity.

### Infection of HiBiT-tagged HBVcc

Generated and purified HiBiT-tagged HBVcc was used to infect human primary hepatocytes or HepG2/NTCP cells in the presence of 4% PEG8000 and 2% dimethyl sulfoxide (DMSO) for 16 hours, as described previously (17). The infection of HiBiT-tagged HBVcc was monitored by measuring HBsAg, HBV DNA, and HiBiT in the culture medium. The HiBiT-tagged HBVcc-infected cells were detected by staining with rabbit polyclonal anti-HBc antibody and Alexa Fluor 555-conjugated anti-rabbit IgG. To confirm that the inserted HiBiT tags in the HBV genome were maintained, the full-length HBV sequence was determined by amplifying the genome of the infected HBV in the culture medium at the end of the observation period using the primer sets described previously (18). As an inhibitor of HBV infection, the preS1 peptide (the equivalent of Myrcludex B), which consists of amino acids 2–48 of the HBV preS1 region of the genotype C strain with amino-terminal myristoylation, was synthesized and used (Scrum Inc., Tokyo, Japan) (9, 19).

### Detection of cccDNA

cccDNA was extracted from HiBiT-tagged HBVcc-infected cells by the Hirt protein-free DNA extraction procedure (20). The cells were treated with SDS and were mixed with a high concentration of NaCl to precipitate high-molecular-weight cellular chromatin and protein bound to DNA. The supernatant containing cccDNA was collected after centrifugation, and cccDNA was extracted with QIAamp DNA Blood Mini Kit (Qiagen). The titer of cccDNA was measured by real-time PCR with a primer and probe set designed to target the cccDNA (9, 16, 21).

## SUPPLEMENTAL MATERIAL

Supplemental Figures msphere.00518-24-s0001.pdf Figures S1 to S3.

ASM does not own the copyrights to Supplemental Material that may be linked to, or accessed through, an article. The authors have granted ASM a non-exclusive, world-wide license to publish the Supplemental Material files. Please contact the corresponding author directly for reuse.
