# Supplementary material for: Exploring the tolerable region for HiBiT tag insertion in the hepatitis B virus genome
Source: mSphere. 2024 Sep 30;9(10):e00518-24. doi: 10.1128/msphere.00518-24 (PMC11520284; doi:10.1128/msphere.00518-24)
Supplement: Supplemental Figures — Figures S1 to S3. [file msphere.00518-24-s0001.pdf]

Supporting Information

Exploring the Tolerable Region for HiBiT Tag Insertion in the Hepatitis B Virus Genome

Running title: Infection System of HiBiT-tagged HBV

Asako Murayama, Hitomi, Igarashi, Norie Yamada,

Hussein Hassan Aly, Masaaki Toyama, Masanori Isogawa,

Tetsuro Shimakami, Takanobu Kato.

Supplementary Figure S1

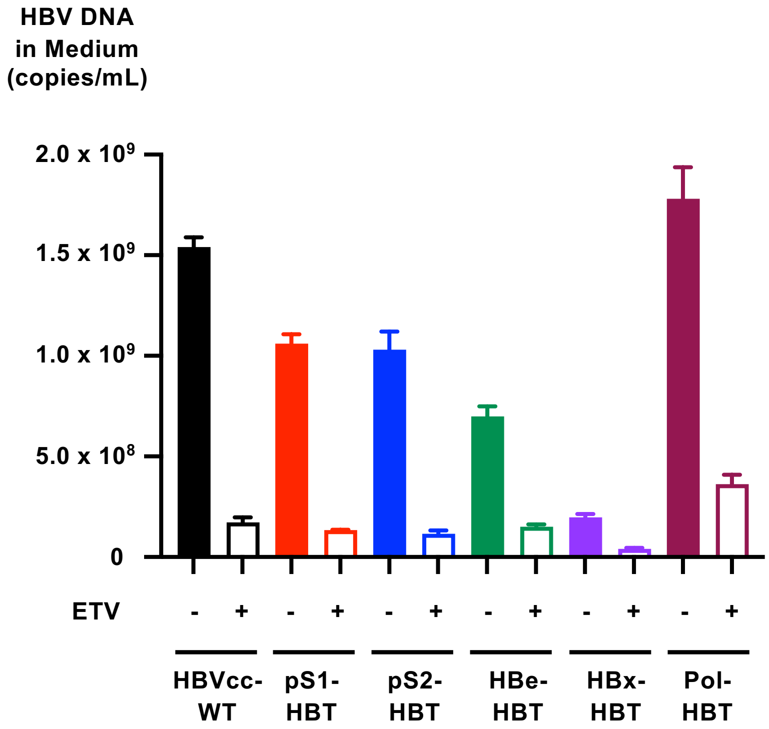

Supplementary Figure S1. Effect of entecavir on production of HBVcc-WT and HiBiT-tagged HBVcc.

HBVcc-WT and HiBiT-tagged HBVcc plasmids were transfected into HepG2/NTCP cells, which were then treated with 100 nM concentration of ETV for 6 days. HBV DNA in the culture medium was measured by real-time PCR using primer and probe sets designed to target the HBs region after DNase treatment and DNA extraction.

Supplementary Figure S2

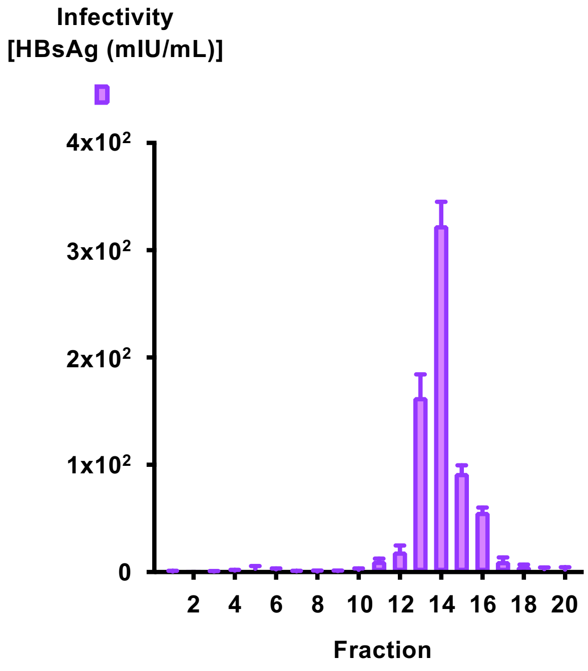

Supplementary Figure S2. Infectivity of HBVcc-WT in fractions of Iodixanol density gradient.

HBVcc-WT in the culture medium of transfected cells was concentrated and purified by an iodixanol density gradient. The infectivity of HBVcc-WT in fractions was evaluated by infection of the same volume of fractions into HepG2/NTCP cells.

Supplementary Figure S3

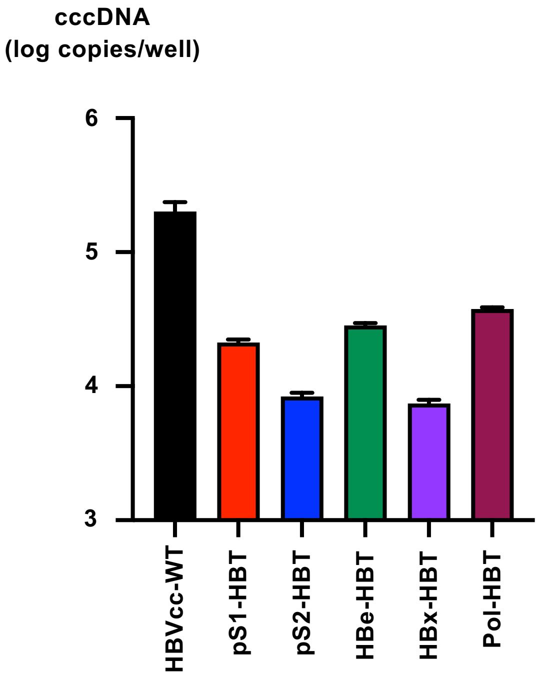

Supplementary Figure S3. Detection of cccDNA in HBVcc-WT and HiBiT-tagged HBVcc-infected cells.

HepG2/NTCP cells were infected with HBVcc-WT and HiBiT-tagged HBVcc at 200 GEq/cell. cccDNA was extracted from infected cells by the Hirt protein-free DNA extraction procedure 12 days after infection and measured by real-time PCR with a primer and probe set designed to target the cccDNA.
